# A Comprehensive Review of Green Hydrogen Technology: Electrolysis Methods, Topologies and Control Strategies, Applications

**DOI:** 10.3390/ma18214826

**Published:** 2025-10-22

**Authors:** Ailitabaier Abudureyimu, Ayiguzhali Tuluhong, Qingpu Chang, Feng Wang, Bao Luo

**Affiliations:** 1 School of Electrical Engineering, Xinjiang University, Urumqi 830047, China; 107552404411@stu.xju.edu.cn (A.A.); 107552304482@stu.xju.edu.cn (Q.C.); 107552404465@stu.xju.edu.cn (F.W.); 107552401472@stu.xju.edu.cn (B.L.); 2 Research Center of Renewable Energy Power Generation and Grid Control Engineering, Ministry of Education, Xinjiang University, Urumqi 830047, China

**Keywords:** green hydrogen, renewable energy, hydrogen production technology, hydrogen power supply

## Abstract

As a pivotal clean energy carrier for achieving carbon neutrality, green hydrogen technology has attracted growing global attention. This review systematically examines four mainstream water electrolysis technologies—alkaline electrolysis, proton exchange membrane electrolysis, solid oxide electrolysis, and anion exchange membrane electrolysis—analyzing their fundamental principles, material challenges, and development trends. It further classifies and compares power electronic converter topologies, including non-isolated and isolated DC–DC converters as well as AC–DC converter architectures, and summarizes advanced control strategies such as dynamic power regulation and fault-tolerant operation aimed at enhancing system efficiency and stability. A holistic “electrolyzer–power converter–control strategy” integration framework is proposed to provide tailored technological solutions for diverse application scenarios. Finally, the challenges and future prospects of green hydrogen across the energy, transportation, and industrial sectors are discussed, underscoring its potential to accelerate the global transition toward a sustainable, low-carbon energy system.

## 1. Introduction

Over the past half century, large-scale exploitation and utilization of fossil fuels have released enormous quantities of carbon dioxide and other greenhouse gases. This has intensified the greenhouse effect and led to profound changes in the global climate system, such as more frequent extreme weather events, rising sea levels, and widespread ecosystem disruption. According to United Nations data, the global average temperature has increased by approximately 0.85 °C over the past 130 years, with the rate of warming in the last three decades reaching an unprecedented level [1]. These changes threaten not only human health and ecological stability but also national energy security [2]. In this context, promoting the transition to clean energy has become both a fundamental approach and the only feasible solution to address the climate crisis [3].

**Figure 1:**
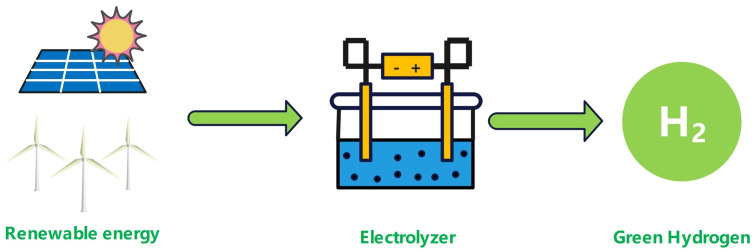
The way to produce green hydrogen.

Hydrogen energy is a storable, electrochemically convertible, and combustible clean energy source characterized by high mass-to-energy density, zero carbon emissions, and broad application potential. These attributes make hydrogen energy crucial for mitigating climate change and establish it as a leading sustainable energy solution for the 21st century [4]. Hydrogen can be produced through various methods. For example, hydrogen obtained via steam methane reforming or gasification is classified as “gray hydrogen.” When steam methane reforming is combined with carbon capture technology, the product is termed “blue hydrogen,” while hydrogen generated through methane pyrolysis is referred to as “turquoise hydrogen.” As shown in Figure 1, hydrogen produced through water electrolysis powered by renewable sources such as solar and wind energy is referred to as “green hydrogen,” an environmentally friendly alternative with a negligible carbon footprint [5].

To date, over 50 countries and regions worldwide have introduced explicit hydrogen energy development strategies and roadmaps, collectively representing a substantial portion of the global economy [6]. The European Union launched A Hydrogen Strategy for a Climate-Neutral Europe in 2020, designating green hydrogen as the core driver of deep decarbonization [7]. In 2023, the United States issued the U.S. National Clean Hydrogen Strategy and Roadmap, aiming to promote emission reduction and strengthen energy security through hydrogen utilization [8]. Other major economies, such as Japan and Australia, have also implemented national hydrogen strategies that set explicit targets for green hydrogen production and export [9,10]. In China, the Medium- and Long-Term Plan for Hydrogen Energy Industry Development (2021–2035) formally integrates hydrogen into the national energy framework, underscoring its strategic importance in future technological and industrial transformation [11,12,13,14,15]. Table 1 summarizes the major national policy documents related to hydrogen energy.

Currently, systematic reviews on green hydrogen production technologies remain limited, and most existing studies focus on isolated aspects such as electrolysis methods, power topologies, or control systems. Therefore, a comprehensive review of current green hydrogen production technologies is essential. This paper first examines, from a materials science perspective, the fundamental principles, material challenges, and development trends of various water electrolysis technologies. Building on this foundation, the paper further proposes a collaborative optimization framework integrating electrolysis technology, power topology, and control strategies for diverse application scenarios. This framework overcomes the limitations of traditional one-dimensional analyses and aims to provide both theoretical foundations and practical guidance for system-level design and performance optimization.

**Figure 2:**
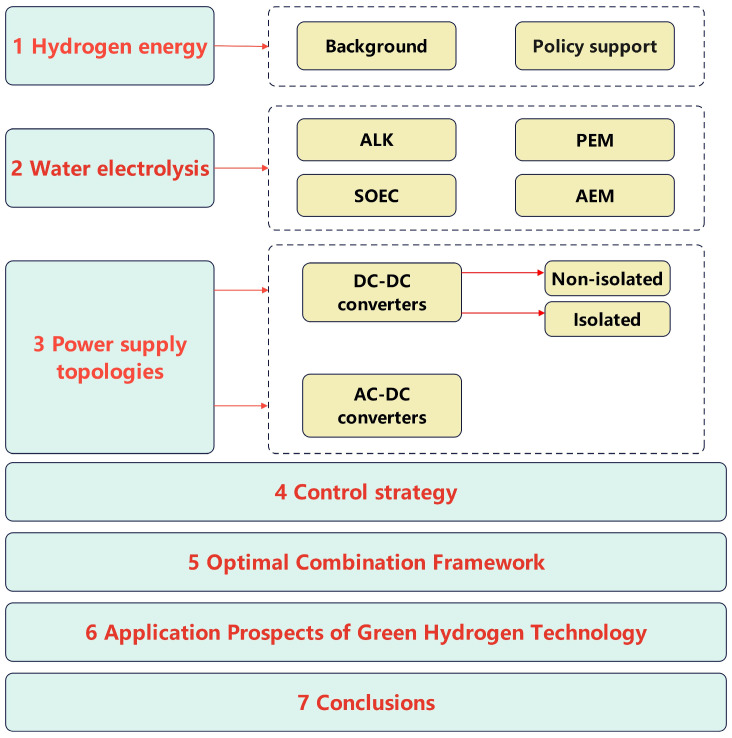
Structure of this review paper.

The structure of this paper is illustrated in Figure 2. The first section presents the overall background of hydrogen energy and reviews relevant domestic and international policies. The second section discusses four methods of hydrogen production via water electrolysis. The third section classifies and analyzes various types of power topologies. The fourth section examines control strategies for water electrolysis-based hydrogen production. The fifth section proposes the concept of an optimal combination framework. Finally, the paper discusses the application prospects of green hydrogen technologies. To aid the reader, Table 2 is provided below, compiling the definitions of technical acronyms, material compositions, and performance metrics that recur in the subsequent sections.

## 2. Hydrogen Production Through Electrolysis of Water

Hydrogen production via water electrolysis employs electrical energy to decompose water into hydrogen and oxygen. This process represents a key technological pathway for producing green hydrogen. Owing to the abundance of water resources and its zero-emission nature, this method is particularly suitable for integration with renewable energy sources (e.g., wind and solar power), facilitating the establishment of a low- or zero-carbon hydrogen supply chain. Depending on the electrolyte type and operating conditions, water electrolysis can be classified into four main categories: Alkaline Electrolysis (AE), Proton Exchange Membrane Electrolysis (PEME), Solid Oxide Electrolysis (SOE), and Anion Exchange Membrane Electrolysis (AEME). Table 3 summarizes the key technical characteristics of these four water electrolysis technologies.

### 2.1. Alkaline Water Electrolysis

**Figure 3:**
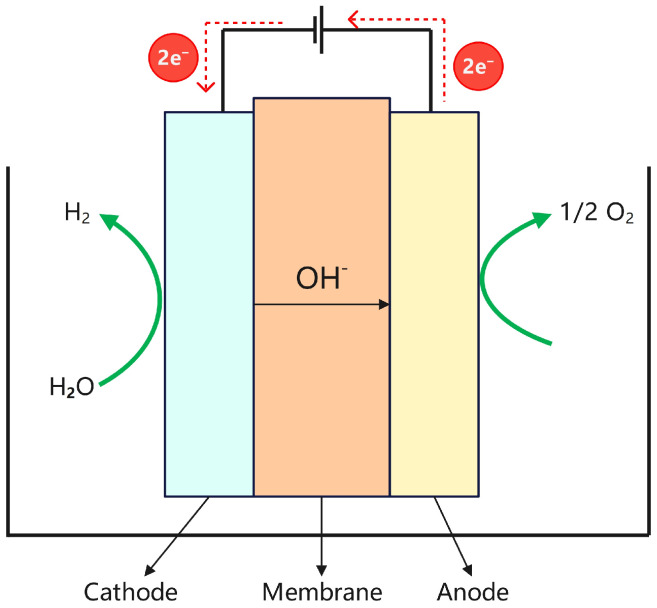
Structure diagram of the ALK electrolyzer.

Alkaline water electrolysis is an electrochemical process that decomposes water into hydrogen and oxygen under the application of an electric current. The electrolyte typically consists of an aqueous solution of potassium hydroxide (KOH) or sodium hydroxide (NaOH). Owing to its technological maturity, cost-effectiveness, and scalability, this method has been widely employed in industrial hydrogen production [18]. As illustrated in Figure 3, hydroxide ions (OH^−^) act as the primary charge carriers within the electrolyte of an alkaline water electrolyzer. The electrolyzer consists of an anode and a cathode, and the corresponding electrochemical reactions are as follows:

Anode: 2OH^−^→1/2O_2_ + H_2_O + 2e^−^, (1)

Cathode: 2H_2_O + 2e^−^→H_2_ + 2OH^−^, (2)

#### 2.1.1. Membrane Material

The physicochemical properties of membrane materials—including electrical resistance, wettability, chemical stability, and mechanical strength—critically influence the system efficiency, gas crossover rate, and operational safety of water electrolyzers. Early industrial electrolyzers typically employed asbestos- or polypropylene-based diaphragms; however, these materials exhibited mechanical embrittlement and poor stability under strongly alkaline conditions. With technological advancement, most commercial alkaline electrolyzers now utilize polymer composite diaphragms primarily filled with zirconia (ZrO_2_), such as Zirfon^®^ and Zirfon PERL. These membranes demonstrate low ionic resistance, outstanding alkaline stability, and superior mechanical flexibility, even in concentrated KOH environments [19].

To further reduce ohmic losses, recent research has focused on enhancing membrane wettability, pore morphology, and ion-channel architecture through surface functionalization and material doping. For example, Peng et al. [20] modified a zirconia-based composite diaphragm using diethanolamine. Under conditions of 25 °C and 1 M KOH, the modified membrane exhibited a water contact angle of 44°, an area resistance of approximately 0.12 Ω·cm^2^, and achieved a current density of 1114 mA cm^−2^ at 2.0 V. Moreover, it maintained stable performance for over 150 h of continuous operation with negligible voltage drift. These findings clearly indicate that optimizing membrane wettability and pore structure can substantially enhance ionic transport within alkaline electrolysis systems.

Nevertheless, under harsh conditions—including concentrated alkaline environments, prolonged operation, and fluctuating temperature or electrolyte concentration—these modified membranes may still experience structural swelling, reduced durability, and increased gas crossover [21]. Therefore, future research should emphasize the evaluation of long-term stability, alkaline corrosion resistance, and gas-separation performance of modified diaphragm materials.

#### 2.1.2. Electrodes and Catalysts

In alkaline water electrolysis, catalytic performance directly governs the hydrogen production rate and overall system efficiency. In recent years, substantial efforts have focused on tailoring catalyst composition and structural design, with particular emphasis on developing non-noble metal catalysts to reduce cost and enhance catalytic activity.

For the hydrogen evolution reaction (HER), cathodes are typically fabricated from Ni, Ni–Mo, or Ni–Fe alloys, while NiFe-LDH, CoFe-LDH, and NiCo_2_O_4_ are commonly used as oxygen evolution reaction (OER) catalysts on the anode. Wang et al. [22] provided a comprehensive overview of recent advances in non-noble metal catalysts for alkaline water electrolysis, emphasizing strategies such as alloying, nano structuring, and surface modification to enhance catalytic efficiency. Numerous studies have demonstrated that Fe-, Ni-, and Co-based catalysts exhibit excellent activity toward the hydrogen evolution reaction (HER). Furthermore, the review highlights the trade-off among stability, activity, and cost, noting that future work should aim to enhance the electrical conductivity and corrosion resistance of catalytic materials. Liu et al. [23] reported a Co_3_Mo_3_N/Co_4_N/Co heterostructure that achieved a cell voltage of only 1.58 V at 10 mA cm^−2^ and exhibited negligible performance degradation after 100 h of continuous operation at 200 mA cm^−2^, demonstrating an excellent balance between catalytic activity and stability. These findings suggest that microstructural engineering of electrodes and optimization of interfacial conductivity are crucial strategies for enhancing the energy efficiency of alkaline electrolyzers.

#### 2.1.3. Electrolyte

In alkaline systems, the electrolyte typically consists of a 20–40 wt% KOH solution, providing an optimal balance between high ionic conductivity and effective gas separation capability. One of the major challenges in alkaline water electrolysis (AWE) arises from the corrosive nature of the electrolyte, which can degrade the performance and durability of the electrodes, membranes, and other cell components.

Electrolyte management also plays a vital role in hydrogen production via alkaline water electrolysis. Optimization of electrolyte concentration, temperature, and pH can significantly improve electrolysis efficiency. Improving electrolyte fluidity and circulation has been shown to reduce energy losses and increase the hydrogen production rate. Brauns et al. [24] developed a dynamic process model consisting of four sub-models to describe the operation of a pressurized alkaline water electrolysis system. These sub-models account for gas contamination, electrolyte concentration, cell voltage, and system temperature. Experimental results demonstrated that appropriate operational strategies and system designs can effectively reduce gas contamination, optimize electrolyte circulation, and enable photovoltaic-powered electrolysis for hydrogen production, thereby maximizing renewable energy utilization and achieving near-zero emissions. However, due to the inherent intermittency of photovoltaic (PV) power, alkaline water electrolysis systems often struggle to adapt to rapid energy fluctuations. Such fluctuations can damage the electrolyzer, increase energy consumption, and shorten the system’s operational lifespan. To address this issue, Cao et al. [25] proposed an integrated hydrogen production system combining PV arrays, battery storage, and alkaline electrolyzers. This system regulates the electrolyzer power output through coordinated electrical control and energy management strategies.

#### 2.1.4. Degradation Pathways

The large-scale commercialization of alkaline water electrolysis (AWE) remains constrained by challenges associated with long-term operational stability. The overall system lifetime is mainly limited by the chemical and electrochemical degradation of critical components. These degradation mechanisms are often interdependent and collectively contribute to the gradual decline in cell performance.

- Membrane aging: Porous composite membranes—such as Zirfon^®^, PPS, and poly (aryl ether) systems—exhibit excellent electrical and mechanical properties. However, under concentrated alkaline conditions and continuous bubble impact, their pore structures may progressively expand, embrittle, or locally collapse, leading to increased gas crossover and higher ohmic resistance [26].
- Reverse-polarity corrosion during shutdown: Repeated start-up and shutdown cycles generate pressure pulsations and thermal expansion mismatches caused by bubble formation, which promote microcrack initiation and reduce membrane durability. Studies have revealed that when the electrolyzer is shut down or experiences sudden load fluctuations, the cathode potential temporarily shifts in the positive direction, causing oxidation of Ni- or NiFe-based catalysts to form NiO layers. This oxidation process reduces the number of active sites and hinders electronic conductivity [27].
- Carbonate deposition and electrolyte contamination: Atmospheric CO_2_ reacts with the KOH electrolyte to form potassium carbonate (K_2_CO_3_) crystals, which may accumulate within membrane pores or flow channels, thereby increasing local resistance and impeding gas diffusion [28]. Consequently, ensuring system sealing integrity, maintaining electrolyte purity, and implementing efficient circulation–purification strategies are essential for extending the operational lifetime of AWE systems.

#### 2.1.5. Future Perspectives

Although alkaline water electrolysis (AWE) is a mature and widely adopted technology for green hydrogen production, its performance is still restricted by limitations in catalyst activity, membrane durability, and gas-separation efficiency. These challenges delineate the development roadmap for the next generation of high-performance alkaline electrolyzers.

- Non-noble metal bifunctional catalysts: Despite significant advances in catalytic activity, these materials continue to suffer from limited stability and structural controllability under high current densities. Future research should transition from single-material development to interface and electronic structure modulation, heterostructure engineering, and the design of self-supported electrodes to simultaneously enhance hydrogen evolution (HER) and oxygen evolution (OER) activities. In addition, integrating in situ characterization techniques with theoretical modeling is essential to elucidate reaction intermediates and deactivation mechanisms, thereby enabling efficient, durable, and cost-effective overall water splitting at high current densities.
- Highly durable hydrophilic composite membranes: Conventional alkaline electrolyzers generally employ Zirfon-type porous composite membranes, which are susceptible to gas crossover and mechanical fatigue under high-pressure or dynamically fluctuating conditions. A promising approach involves incorporating hydrophilic inorganic fillers (e.g., ZrO_2_, TiO_2_, SiO_2_) and fabric-reinforced structures into polymer matrices to enhance hydrophilicity, ionic conductivity, and mechanical robustness. Moreover, precise control of the microporous structure in composite membranes can improve gas–liquid separation and lower ohmic resistance. Future membrane designs should aim to simultaneously achieve high OH^−^ conductivity, low gas permeability, and superior alkaline resistance.
- Gas crossover: Alkaline water electrolysis is generally performed in concentrated KOH or NaOH electrolytes using nickel-based electrodes and relatively thick diaphragms. During electrochemical operation, hydrogen and oxygen gases can permeate or diffuse through the diaphragm or porous separator, resulting in decreased gas purity, reduced energy efficiency, and potential safety risks. To mitigate this problem, reducing membrane thickness and optimizing structural density can effectively suppress gas crossover.

### 2.2. Proton Exchange Membrane Water Electrolysis

**Figure 4:**
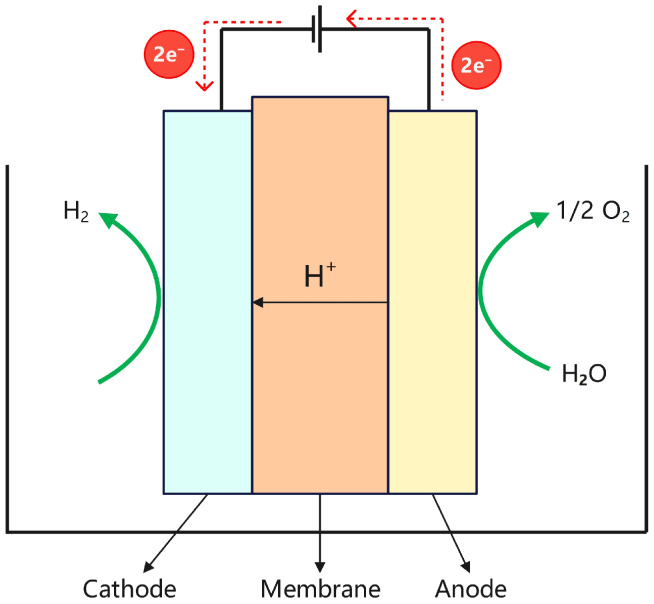
Structure diagram of the PEM electrolyzer.

Proton Exchange Membrane (PEM) water electrolysis is a process that generates hydrogen and oxygen through the electrochemical decomposition of water. A key feature of this system is the use of a proton exchange membrane that simultaneously serves as both the electrolyte and the gas-separation diaphragm. The membrane selectively allows protons (H^+^) to pass while blocking electrons, thereby establishing a potential difference between the electrodes and enabling the electrochemical decomposition of water into hydrogen and oxygen. During electrolysis, water molecules are oxidized at the anode to produce oxygen gas, protons, and electrons. The protons migrate through the proton exchange membrane to the cathode, where they react with electrons to form hydrogen gas via a reduction reaction. Meanwhile, electrons flow from the anode to the cathode through an external circuit, providing the electrical energy required for the reaction. The structural configuration of the PEM electrolyzer is illustrated in Figure 4, and the corresponding electrochemical reactions are as follows:

Anode: H_2_O → 1/2O_2_ + 2H+ + 2e^−^, (3)

Cathode: 2H+ + 2e^−^ → H_2_, (4)

#### 2.2.1. Membrane Material

Regarding membrane materials, the Aquivion^®^ membrane has been increasingly adopted as a substitute for the conventional Nafion^®^ membrane due to its superior chemical stability, lower gas crossover, and higher glass-transition temperature. These features make it particularly suitable for proton exchange membrane water electrolysis (PEMWE) systems operating under high current densities and elevated temperatures. Furthermore, the incorporation of Ce-based radical scavengers has been demonstrated to further improve the durability and electrochemical performance of Aquivion^®^ membranes [29]. In hydrocarbon-based membrane systems, embedding CeO_2_ nanoparticles within interlocking interfacial layers effectively suppresses hydrothermal degradation and prevents mechanical delamination at the interface. As a result, the cell exhibited a voltage rise rate as low as 48 µV h^−1^ over 500 h of operation—significantly lower than that of the Nafion-based benchmark [30]. In terms of membrane electrode assembly (MEA) design, Stiber et al. [31] reported that a Nb/Ti-coated porous structural configuration effectively enhanced gas removal efficiency and promoted uniform water distribution, thereby optimizing two-phase flow behavior within the reaction zone. Their study demonstrated that this configuration significantly reduced cell overpotential and improved overall electrolyzer efficiency, exhibiting superior performance, particularly under high-current-density operation.

#### 2.2.2. Electrodes and Catalysts

The oxygen evolution reaction (OER) occurring at the anode depends on highly efficient catalysts. Among them, iridium oxide (IrO_2_) and platinum (Pt) are the most widely used catalysts in proton exchange membrane electrolysis cells (PEMECs) due to their outstanding catalytic activity and superior stability. However, their high cost poses a major obstacle to the large-scale commercialization of PEMEC technology [32].

Li et al. [33] successfully synthesized a hierarchically porous IrO_2_ nano catalyst with combined microporous and mesoporous architectures using an ammonia-induced templating method. The resulting catalyst exhibited a uniform pore-size distribution and, compared with conventional IrO_2_, showed a 30 mV reduction in overpotential at 10 mA·cm^−2^, indicating enhanced intrinsic OER activity. Perovskite-type oxides (ABO_3_ structures), in which A- and B-site cations can be substituted, provide a versatile platform for tuning the chemical environment and electronic structure of active metal sites, thereby optimizing OER activity [34]. By varying the A- and B-site compositions, the electronic configuration and lattice oxygen content can be precisely modulated to enhance catalytic performance. Kumar et al. [35] synthesized FeCoP nanorods through a facile one-pot colloidal method, which exhibited remarkable OER activity in 1.0 M KOH, achieving a current density of 50 mA·cm^−2^ at an overpotential of only 230 mV and maintaining excellent stability over 60 h of continuous operation.

#### 2.2.3. Modeling and Simulation

As proton exchange membrane water electrolysis (PEMWE) systems become increasingly complex, researchers have developed multidimensional and multiphysics models to capture the coupled effects of electrochemical reaction kinetics, heat and mass transfer, bubble dynamics, and flow-field interactions. Among these, two- and three-dimensional computational fluid dynamics (CFD) models are widely employed to optimize anode geometries and water transport pathways [36]. Furthermore, artificial intelligence (AI) and machine learning (ML) techniques have been incorporated into PEMWE research to enhance modeling efficiency and predictive accuracy, particularly for system lifetime estimation and fault diagnosis [37]. For example, Rahul Kumar et al. [38] employed a shallow long short-term memory (LSTM) neural network to diagnose open-circuit faults in the power-switch components of PEM electrolyzer power systems. The model successfully detected and localized faults at each time step without the need for data preprocessing, demonstrating the strong potential of data-driven approaches for real-time system monitoring and control.

#### 2.2.4. Degradation Pathways

Proton exchange membrane (PEM) electrolyzers—characterized by high current densities, compact stack designs, and rapid dynamic response—are considered among the most promising technologies for renewable hydrogen production. However, prolonged operation under strongly acidic conditions and high anodic potentials leads to multiscale material degradation and structural evolution.

- Dissolution and migration of noble-metal catalysts: At anodic potentials above 1.6 V (vs. RHE), Ir or IrO_2_ catalysts can partially dissolve to form soluble Ir^4+^ species, which subsequently migrate into the membrane or redeposit onto the cathode under the influence of the electric field. This “dissolution–migration–redeposition” cycle gradually reduces the electrochemically active surface area (ECSA) [39]. Moreover, fluctuations in current density and start–stop cycling accelerate the dissolution process, resulting in a nonlinear accumulation of performance degradation.
- Chemical degradation of the proton exchange membrane: Under highly oxidative conditions, perfluoro sulfonic acid (PFSA) membranes generate H_2_O_2_ and hydroxyl radicals that attack the polymer backbone and side chains, leading to fluoride release, membrane thinning, and reduced proton conductivity. This degradation is manifested by increased ohmic resistance and enhanced gas crossover, which may ultimately cause localized short circuits [40].
- Degradation of titanium bipolar plates: The titanium bipolar plates on the anode side are particularly susceptible to corrosion-induced degradation. In acidic and high-potential environments, fluoride ions (F^−^) generated from membrane decomposition can compromise the stability of the passive TiO_2_ layer, leading to elevated interfacial contact resistance and localized corrosion [41].

#### 2.2.5. Future Perspectives

Compared with alkaline water electrolysis, proton exchange membrane (PEM) technology exhibits significant advantages in terms of high current density and compact system design. However, further commercial expansion requires a strong focus on cost reduction and overall performance enhancement. The key research priorities in this regard include:

- Proton exchange membranes: Although extensive research has been conducted on alternative materials, no single membrane has yet matched Nafion^®^ in terms of overall performance and durability. Future research should focus on developing next-generation proton exchange membranes with high proton conductivity, exceptional chemical stability, and lower cost to overcome the limitations of Nafion^®^. Core directions include: optimizing short-side-chain perfluoro sulfonic acid membranes to achieve superior performance and mechanical strength at elevated operating temperatures; exploring non-fluorinated or partially fluorinated hydrocarbon polymers, combined with cross-linked structures or nanofiller incorporation to mitigate chemical degradation under harsh operating conditions while reducing cost and environmental impact; and designing multifunctional composite membranes by introducing inorganic nanoparticles to simultaneously enhance water retention, mechanical robustness, and radical scavenging capability.
- Development of corrosion-resistant conductive coatings: To achieve safe, efficient, and long-lifespan PEM water electrolysis (PEMWE) operation under high-pressure conditions, developing high-performance corrosion-resistant and conductive coatings is a critical engineering objective. Future studies should aim to replace expensive noble-metal coatings with highly stable material systems such as transition-metal nitrides, carbides, and conductive oxides. Advanced surface engineering techniques are required to fabricate dense, uniform, and strongly adherent thin-film coatings on titanium-based bipolar plates and porous transport layers.
- Integration of PEMECs with renewable energy systems: The key objective is to elucidate the degradation mechanisms of electrolyzers under dynamically fluctuating power conditions and, based on these insights, develop intelligent system control strategies and lifetime management methods. These include optimizing start-up and shutdown protocols, expanding the operational range for rapid load variations, and establishing hybrid systems integrated with energy storage devices to mitigate power fluctuations. The ultimate goal is to develop an integrated system capable of responding in real time to renewable energy inputs while maximizing energy efficiency and device lifetime, thereby positioning PEMECs as efficient, flexible, and reliable “green hydrogen” production units within future power infrastructures.

### 2.3. Solid Oxide Electrolysis

**Figure 5:**
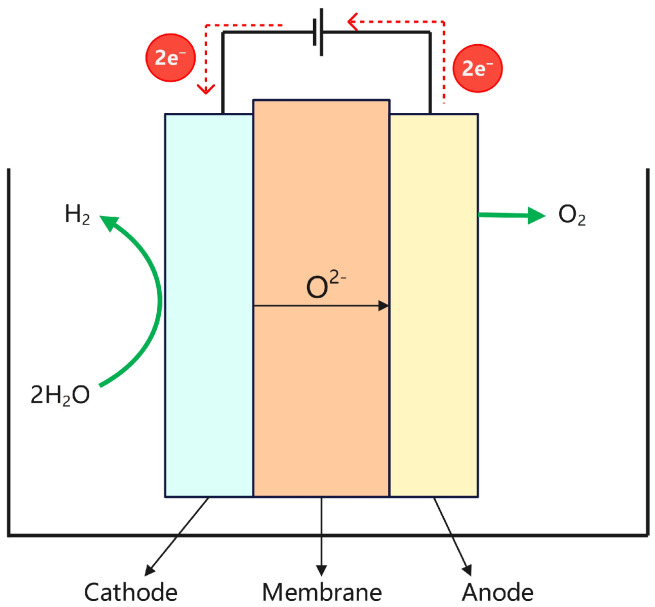
Structure diagram of the SOE electrolyzer.

Solid Oxide Electrolysis Cells (SOECs) generate hydrogen by decomposing water at high temperatures. Since first being reported by Dönitz and Erdle in the 1980s, SOECs have attracted considerable attention due to their low electrolysis voltage and high Faradaic efficiency. SOEC technology operates at high temperatures (typically 500–1000 °C), offering superior electrochemical performance and efficiency compared with alkaline and PEM electrolyzers [42,43]. However, it remains primarily at the experimental research stage. Based on their technical principles, SOECs are classified in-to oxygen-ion-conducting and proton-conducting types. The basic principle of SOEC hydrogen production is that high-temperature steam enters the cell and decomposes at the cathode into H^+^ and O^2−^ ions. The H^+^ ions gain electrons and form hydrogen (H_2_), while the O^2−^ ions migrate through the electrolyte to the anode, where oxygen (O_2_) is released. The structure of the electrolyzer is shown in Figure 5, and the specific electrochemical reactions of this process are as follows:

Anode: O^2−^ → 1/2O_2_ + 2e^−^, (5)

Cathode: H_2_O + 2e^−^ → H_2_ + 2O^2−^, (6)

#### 2.3.1. Electrolyte

The electrolyte in solid oxide electrolysis cells (SOECs) must be a dense oxygen-ion conductor that exhibits high ionic conductivity, minimal electronic leakage, excellent chemical stability, and strong mechanical compatibility at elevated temperatures. Currently, the most widely used electrolyte material is yttria-stabilized zirconia (YSZ)—a solid solution of zirconium dioxide (ZrO_2_) containing approximately 15 mol% yttrium oxide (Y_2_O_3_) [44]. YSZ exhibits high ionic conductivity and excellent chemical stability, enabling stable oxygen-ion transport at elevated operating temperatures. The 8 mol% yttria-stabilized zirconia (8-YSZ) composition is widely employed as an oxide-ion electrolyte in high-temperature solid oxide fuel cells (HT-SOFCs), due to its ionic conductivity of approximately 0.1 S·cm^−1^ at 1000 °C. However, the primary limitation of YSZ is its sharp decline in ionic conductivity at intermediate and low temperatures (<800 °C), which significantly limits the operational efficiency and economic feasibility of SOEC systems [45]. To lower the operating temperature, researchers have investigated a variety of alternative electrolyte materials. Among these, scandia-stabilized zirconia (ScSZ) typically exhibits higher ionic conductivity than YSZ at comparable temperatures. However, the high cost of Sc_2_O_3_ precursors and potential phase instability during long-term operation have limited its large-scale implementation [46].

#### 2.3.2. Electrodes and Catalysts

In solid oxide electrolysis cells (SOECs), electrode materials must exhibit high electronic conductivity, partial ionic conductivity, excellent catalytic activity, and outstanding thermochemical stability. Conventional Ni–YSZ (nickel–yttria-stabilized zirconia) fuel electrodes have been extensively modified through nanoengineering techniques such as infiltration, atomic layer deposition (ALD), and pulsed laser deposition (PLD) [47,48,49]. These techniques reduce the catalyst particle size from the micrometer to the nanometer scale, thereby significantly increasing the triple-phase boundary (TPB) length and enhancing both catalytic activity and long-term durability.

In recent years, perovskite-type and multicationic oxides (e.g., La–Sr–Co/Fe systems, LST, and LSCrM) have been explored as reconfigurable or alternative fuel electrodes to achieve improved oxidation and carbon tolerance, along with enhanced structural self-reconstruction capability. Conversely, typical oxygen electrode (anode/OER-side) materials include LSM, LSCF, and their composite systems. Among them, LSCF (La_0_._6_Sr_0_._4_Co_0_._2_Fe_0_._8_O_3–δ_) is widely employed at intermediate-to-high temperatures owing to its high oxygen reduction and evolution activities. However, its long-term stability is limited by factors such as Sr surface segregation, interfacial reactions with the electrolyte, and chromium poisoning [50].

To address these challenges, researchers have adopted strategies including A/B-site doping, surface modification, interfacial buffer layer construction, and the incorporation of multi-element (high-entropy) oxide electrodes to suppress cation segregation and phase transformation, thereby improving structural stability and operational lifetime. Overall, the central challenge in electrode material design lies in achieving an optimal balance between catalytic activity and stability while maintaining compatibility with both the electrolyte and interfacial architecture.

#### 2.3.3. Degradation Pathways

During prolonged operation of solid oxide electrolysis cells (SOECs), complex degradation phenomena occur across multiple structural levels, including changes in electrode composition, interfacial integrity, and interconnect stability.

- Migration and oxidation of Ni-based electrodes: In the fuel electrode (cathode), nickel particles are oxidized to form volatile Ni (OH)_x_ species under high steam concentrations during electrolysis, which subsequently migrate toward the electrolyte surface. This process increases the ohmic resistance, reduces the triple-phase boundary (TPB) length, and consequently degrades the catalytic performance of the electrode [51]. Furthermore, the formation of NiO further decreases both the surface catalytic activity and electronic conductivity of the electrode. Studies have also revealed that Ni migration is more pronounced during CO_2_ electrolysis than in steam electrolysis [52]. To mitigate Ni oxidation and enhance the electrode’s electrochemical performance, strategies such as introducing reducing gases or incorporating metal dopants are commonly adopted.
- Delamination and thermally induced cracking at the anode/electrolyte interface: During electrolysis mode, the oxygen electrode (anode) operates under high oxygen partial pressure, where localized gas accumulation and thermal expansion mismatch may lead to bubble formation and mechanical delamination at the electrode–electrolyte interface.
- Corrosion of interconnects and accumulation of interfacial resistance: The interconnects in SOEC stacks are generally fabricated from stainless steel. At elevated temperatures, volatile chromium species such as CrO_2_(OH)_2_ decompose into Cr_2_O_3_ and related oxides, which subsequently deposit on the surfaces of the electrodes and electrolytes. These chromium oxide deposits increase polarization resistance, thereby contributing to the gradual degradation of SOEC performance [53]. In a 25 kW-class SOEC stack operated continuously for 4000 h, Lang et al. [54] reported an average degradation rate of approximately 2.8% per 1000 h, primarily attributed to oxide-layer thickening on interconnect surfaces and increased interfacial contact resistance.

#### 2.3.4. Scaling-Up and Commercialization

In terms of scale-up and commercialization, solid oxide electrolysis cell (SOEC) technology has achieved remarkable progress in recent years. Typical SOEC stacks possess active electrode areas of approximately 100 cm^2^, while larger stacks with areas up to 500 cm^2^ have been successfully fabricated and demonstrated [55]. Léon et al. [56] analyzed the performance degradation of SOEC stacks during prolonged operation. Their results indicated that enhancements in material structure and sealing technology are crucial for achieving stable large-scale operation. The study further emphasized the importance of system integration and the optimization of operating parameters. On the commercialization front, several demonstration projects have promoted the transition of SOEC technology from laboratory research to industrial-scale application. For instance, the SOEC demonstration plant in Salzgitter, Germany, achieved stable operation of a 40 Nm^3^ H_2_ h^−1^ hydrogen production system, providing reliable data to support the industrial deployment of green hydrogen. Larger-scale SOEC plants are expected to be commissioned in the coming years, further advancing the commercialization of this technology [57].

#### 2.3.5. Future Perspectives

To advance the transition of solid oxide electrolysis cell (SOEC) technology from laboratory research to large-scale application, two critical challenges must be addressed: enhancing long-term durability under high-temperature and harsh operating conditions, and developing economically viable process integration pathways.

- Durability: In solid oxide electrolysis (SOE/SOEC) systems, the primary technical challenges involve maintaining high-temperature material stability and stack-level durability under harsh operating conditions. For example, Ni–YSZ cathodes are susceptible to Ni migration, phase transformation, and particle agglomeration during high-temperature steam electrolysis or under CO_2_ co-feeding conditions, leading to a reduction in the active triple-phase boundary region. Additionally, chromium poisoning, oxide scale growth, and interfacial resistance accumulation at the interface between the air electrode and metallic interconnects are key factors that limit stack lifetime. To advance the commercialization of SOEC technology, it is essential to achieve low degradation rates under high current densities and extended operation, while developing comprehensive mitigation strategies integrating material design, atmosphere control, and interface engineering.
- Process integration: Another promising approach is to couple SOECs with other chemical synthesis processes—such as methane-to-ethylene conversion or nitrogen-to-nitric oxide synthesis—to enhance the overall techno-economic viability of SOEC technology. Such hybrid systems enable energy-efficient co-production of hydrogen and value-added chemicals, thereby enhancing overall energy utilization efficiency and commercial competitiveness.

### 2.4. Anion Exchange Membrane Electrolysis

Anion Exchange Membrane Water Electrolysis (AEMWE) is an emerging technology that integrates features of traditional Alkaline Water Electrolysis (ALK) and Proton Exchange Membrane Water Electrolysis (PEMWE) [58]. This technology has the advantages of lower cost, higher performance, adjustable electrolyte pH, lower electrolyte concentration requirement, and higher hydrogen yield. However, challenges remain, particularly regarding membrane stability and electrode corrosion in alkaline environments. Consequently, ongoing research seeks to enhance AEMWE performance and stability, thereby facilitating its broader application in hydrogen production and related fields.

**Figure 6:**
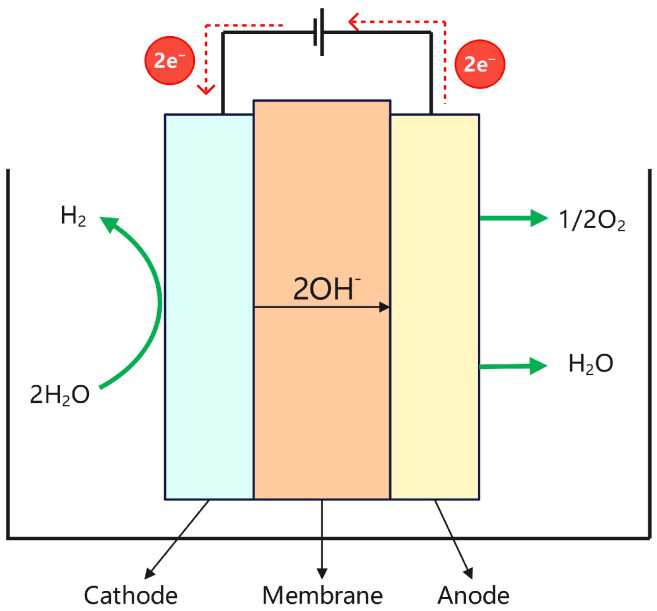
Structure diagram of the AEM electrolyzer.

The main components of an AEM hydrogen electrolyzer include the AEM, catalyst layer (CL), gas diffusion layer (GDL), bipolar plate, and end plate [59]. Its electrolyzer structure is shown in Figure 6, and the alkaline water decomposition reaction and thermodynamic reaction equation are as follows:

Anode: 2OH^−^ → 1/2O_2_ + H_2_O + 2e^−^, (7)

Cathode: H_2_O + e^−^ → 1/2H_2_ + OH^−^, (8)

#### 2.4.1. Membrane Material

The anion exchange membrane (AEM) is a key component of an AEM water electrolyzer, serving two essential functions:

1. Providing an internal pathway for hydroxide (OH^−^) ion conduction
2. Separating the hydrogen and oxygen gases produced at the cathode and anode, respectively, to mitigate potential safety risks.

Conventional quaternary ammonium (QA)-functionalized polymers—such as poly (arylene ether) s, polybenzimidazoles, polysulfones, and PPO-based materials—exhibit relatively high ionic conductivity. However, under alkaline and oxidative conditions, these polymers are susceptible to SN2 substitution, Hofmann elimination, and main-chain scission or oxidative degradation, resulting in structural breakdown and loss of ionic conductivity [60]. Yin et al. [61] developed a novel AEM incorporating quinuclidinium-based cationic side chains and a branched polyarylene backbone (PAQ-x), achieving a favorable balance of high ionic conductivity, low dimensional swelling, and excellent alkaline and thermal stability. Specifically, the PAQ-5 membrane achieved a current density of 8 A cm^−2^ in non-noble-metal-catalyzed AEM water electrolysis (AEMWE), maintaining stable operation for more than 2400 h under gradient current conditions. Beyond the PAQ-x series, substantial progress has been achieved in AEM structural design, particularly through the introduction of rigid microporous channels that facilitate continuous OH^−^ transport while preserving chemical robustness. For instance, a heteroatom-free microporous framework (HFM) reported by Wang et al. exhibited an outstanding hydroxide conductivity of 215 mS cm^−1^ at 80 °C, together with remarkable alkaline durability, retaining structural integrity after more than 3000 h of immersion in 1 M KOH solution [62].

#### 2.4.2. Electrodes and Catalysts

To fully realize the cost advantage of anion exchange membrane water electrolysis (AEMWE), the development of non-noble metal catalysts is crucial for reducing overall system cost.

In the hydrogen evolution reaction (HER), transition-metal alloys—particularly Ni–Mo-based catalysts—have been extensively investigated and demonstrated great potential. Liu et al. [63] reported that a MoNi_4_/MoO_2_ catalyst, composed of MoNi_4_ nanoparticles anchored on a MoO_2_ substrate and synthesized via hydrothermal and annealing processes, required only ~55 mV of overpotential to achieve a current density of 500 mA·cm^−2^ in 1.0 M KOH, with a Tafel slope of ~57 mV·dec^−1^, comparable to that of commercial Pt/C catalysts. This superior activity was attributed to the synergistic effect between Mo, which facilitates H_2_O dissociation, and Ni, which provides an optimal H* adsorption energy. Another study further confirmed the high efficiency of Ni–Mo alloys in practical AEMWE systems [64].

In the oxygen evolution reaction (OER), multicomponent transition-metal oxides and hydroxides (e.g., Ni, Co, and Fe systems) have attracted considerable attention due to their tunable electronic structures and abundant active sites. For example, Cu-doped Co_3_O_4_ spinel oxides (Cu_x_Co_3−X_O_4_) have been identified as highly efficient OER catalysts. Previous studies have shown [65,66] that incorporating Cu into Co-based hydroxides effectively modulates the local electronic environment of Co, thereby optimizing the adsorption free energy of key oxygen evolution intermediates. Consequently, certain Cu–Co hydroxide catalysts exhibit outstanding oxygen evolution reaction (OER) activity in alkaline media, achieving current densities up to 500 mA cm^−2^ with overpotentials as low as ~420 mV in 1.0 M KOH. In addition, ternary Ni–Co–Fe (hydro)oxides have emerged as highly promising OER electrocatalysts. Their amorphous or defect-rich structures provide abundant active sites, and under alkaline conditions, they often demonstrate both higher activity and greater durability than the benchmark IrO_2_ catalyst.

#### 2.4.3. Degradation Pathways

Current studies have revealed that the key degradation pathways limiting the lifetime of anion exchange membrane water electrolysis (AEMWE) systems originate from the intrinsic chemical instability of the membrane and catalyst layers, as well as the electrochemical incompatibility among heterogeneous interfaces within the cell.

- Chemical degradation: In the strongly alkaline operating environment, the anion exchange membrane (AEM) is the component most susceptible to chemical attack and degradation. The cationic functional groups (e.g., quaternary ammonium sites) attached to the polymer backbone are vulnerable to nucleophilic attack by hydroxide ions (OH^−^), initiating SN2 substitution and Hofmann elimination reactions that irreversibly deactivate ionic conduction sites. Studies have shown that replacing conventional benzyl trimethylammonium groups with piperidinium or sterically hindered cations (e.g., bicyclic or spirocyclic structures) can effectively resist OH^−^ attack through steric protection mechanisms. A systematic screening study [67] confirmed the superior alkaline stability of these cationic groups. Moreover, polymer backbones containing ether linkages are susceptible to base-catalyzed hydrolysis, leading to chain scission, decreased mechanical strength, and loss of ionic conductivity. This bulk degradation process, propagating from the functional groups to the polymer backbone, constitutes the primary limitation to the long-term durability of AEMs. A study [68] further demonstrated that all-carbon backbones (e.g., SEBS) exhibit superior alkaline stability compared with ether-containing backbones (e.g., PSU).
- Interfacial degradation: This arises from physicochemical mismatches at the interfaces between different components. Within the catalyst layer, the ionomer not only undergoes chemical degradation but also adsorbs strongly onto the catalyst surface via its aromatic backbone, blocking active sites and substantially hindering hydrogen evolution reaction (HER) kinetics. To mitigate the adsorption of aromatic rings on catalyst surfaces, ref. [69] reported an aliphatic-backbone ionomer that effectively reduced site blockage and enhanced both reaction kinetics and operational stability. Furthermore, the formation of oxide or corrosion layers on bipolar plates and gas diffusion layers under high anodic potentials markedly increases interfacial contact resistance, thereby accelerating overall system degradation.

#### 2.4.4. Future Perspectives

AEM water electrolysis represents a promising next-generation hydrogen production technology, yet its limited long-term stability remains a critical barrier to practical deployment.

- Anion exchange membranes: The development of AEMs with high ionic conductivity, robust mechanical strength, and superior chemical stability is critical to overcoming the current performance bottlenecks in anion exchange membrane water electrolysis (AEMWE). A deeper understanding of the degradation mechanisms of both the polymer backbone and cationic functional groups is therefore essential, along with the rational design of highly stable cationic sites and optimized backbone/side-chain architectures to simultaneously enhance ionic conductivity and long-term durability.
- Elucidation of degradation and failure mechanisms: A comprehensive understanding of the degradation and failure mechanisms of AEMs is fundamental for achieving long-term operational stability and facilitating the industrial deployment of AEMWE systems. Despite notable progress in ionic conductivity and non-noble-metal catalyst compatibility, AEMs still exhibit inferior chemical stability, mechanical integrity, and interfacial durability compared with PEM and alkaline electrolysis technologies. During prolonged operation, AEMs are susceptible to chemical degradation, loss of ionic conductivity, and interfacial delamination, which collectively lead to voltage rise, gas crossover, and reduced stack lifetime. Therefore, identifying the intrinsic causes of degradation and lifespan limitations is vital for the development of commercially viable and high-performance AEM-based electrolyzers.
- Development of high-performance electrolyzer components: The overall efficiency, durability, and safety of an AEMWE system depend on the synergistic performance of all cell components, rather than on the membrane alone. Beyond membrane optimization, further progress is required in cathode and anode catalysts, ionomers, and the integration of membrane electrode assemblies (MEAs). Current studies have primarily focused on AEMs and electrocatalysts, whereas interfacial phenomena—particularly those at the catalyst–membrane and catalyst–porous transport layer interfaces—remain insufficiently understood. A systematic investigation of these interfacial phenomena is essential to improving MEA performance and ensuring long-term operational reliability.

## 3. Topologies of Hydrogen Power Supply

The use of power electronic converters, i.e., hydrogen power supplies, has become indispensable in view of the electrical specifications required for the operation of industrial electrolyzers [70]. Such converters play a vital role in ensuring the safe and efficient operation of the electrolyzer, and if they do not work stably, they can affect the purity and hydrogen production rate of the hydrogen produced by the system. In order to meet the demands of the electrolyzer, the power electronic converter is required to have characteristics such as low output current ripple, high current carrying capacity, high dropout ratio, wide output voltage range and high reliability.

**Figure 7:**
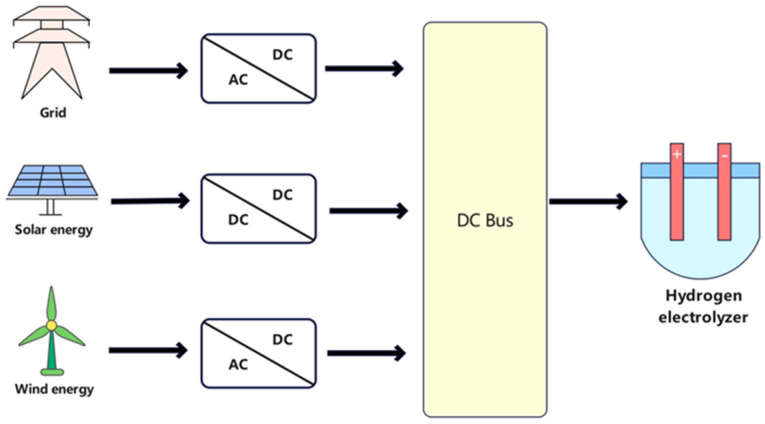
Power electronics converters for different energy sources in electrolyzer applications.

As shown in Figure 7, in order to meet the conditions for electrolyzer operation, when the electrolyzer is powered by the grid or wind farm, uses an AC-DC converter to convert the AC power to DC power that meets the voltage specification of the electrolyzer. If the electrolyzer is powered by solar panels, a DC-DC converter is used.

### 3.1. DC-DC Converters

#### 3.1.1. Non-Isolated DC-DC

**Figure 8:**
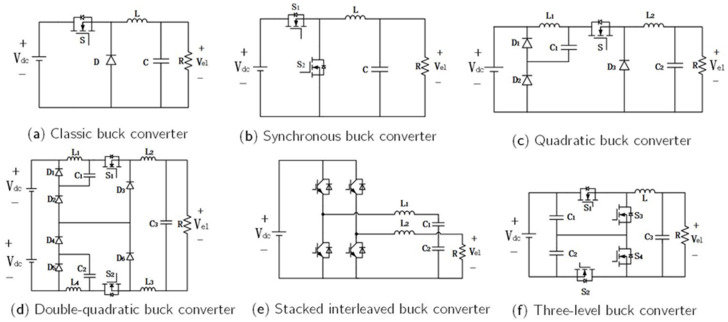
Non-isolated DC-DC converters for electrolyzer applications.

Non-isolated converters are predominantly derived from the Buck topology [71]. The classic Buck converter (Figure 8a) is one of the most widely used power conversion circuits, applied in electrolyzer power supplies because of its simple structure, low cost, ease of control, and reliable step-down capability. However, the voltage conversion ratio of a classic Buck converter is inherently limited. When a low output voltage is required, the duty cycle becomes extremely small, causing the inductor current to operate in discontinuous conduction mode (DCM). Efforts to suppress current ripple inevitably result in higher switching losses and larger inductors, thereby limiting the use of conventional Buck converters as power supplies for water electrolysis systems [72].

Sahin et al. [73] proposed a synchronous Buck converter (Figure 8b) in which the diode was replaced with a MOSFET to reduce conduction losses and improve conversion efficiency. This configuration is particularly suitable for systems demanding high energy efficiency. To achieve a higher voltage conversion ratio, Guilbert et al. [74] implemented a quadratic Buck converter (Figure 8c), incorporating current-controlled sliding-mode control to satisfy the dynamic response requirements of PEM electrolyzer hydrogen production systems. De et al. [75] introduced a double-quadratic Buck converter (Figure 8d), which reduced voltage stress on power switches while maintaining equivalent step-down performance. The interleaved Buck converter (Figure 8e) [76] has been widely adopted in medium- and low-power PEM electrolyzer systems owing to its low output current ripple, high efficiency, and superior redundancy. Compared with the conventional two-level Buck converter, the three-level Buck topology (Figure 8f) reduces the voltage stress on each power switch to half of the input voltage [77]. This topology enables low-voltage, high-current output, making it suitable for electrolysis systems operating under high DC-bus voltages.

Beyond the aforementioned configurations, ref. [78] summarized several additional non-isolated DC–DC converter topologies, including tapped-inductor Buck converters, Buck converters with switched-inductor modules, and hybrid switched-capacitor/switched-inductor Buck converters. Although these variants exhibit enhanced performance relative to the conventional Buck converter, their structural complexity and limited applicability hinder widespread adoption in practical hydrogen production systems.

#### 3.1.2. Isolated DC-DC

Isolated DC–DC converters are indispensable in electrolyzer applications, characterized by electrical insulation between input and output, typically achieved through high-frequency transformers. These converters are generally categorized into single-stage, two-stage, parallel, and multi-port configurations [79]. With the rapid development of hydrogen production power systems, the performance requirements for converters have become increasingly stringent.

**Figure 9:**
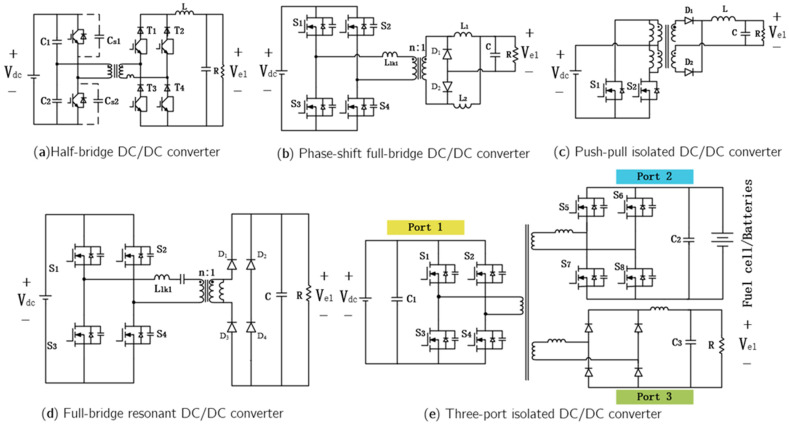
Isolated DC-DC converters for electrolyzer applications.

In large-scale hydrogen production systems, half-bridge and full-bridge isolated DC–DC converters are most commonly employed. The half-bridge DC–DC converter topology (Figure 9a) features low switching losses and a wide voltage regulation range. Under the wide input voltage conditions typical of renewable energy systems, it can further enhance electrolyzer hydrogen production efficiency [80]. The full-bridge DC–DC converter topology suitable for photovoltaic (PV)-based hydrogen production systems is shown in Figure 9b. This converter achieves zero-voltage switching (ZVS) and exhibits low output current ripple and stable output voltage, enabling high circuit efficiency and stable operation in PV-powered hydrogen production systems [81].

Garrigós et al. [82] proposed a push–pull isolated DC–DC converter powered by photovoltaic panels (Figure 9c). Its advantages include a simple driving circuit, elimination of bulky and expensive voltage-balancing capacitors, and control through a straightforward PWM scheme. However, this topology suffers from large hard-switching losses, high voltage stress on primary-side devices, and complex center-tapped transformer structures. It is therefore more suitable for cost-sensitive hydrogen production applications with moderate efficiency requirements.

Ref. [83] introduced a full-bridge resonant converter topology for PEM electrolyzers by incorporating a resonant circuit into the transformer primary side. As shown in Figure 9d, this topology offers high efficiency, achieves zero-voltage switching over the full load range, eliminates duty-cycle loss, and suppresses rectifier oscillations without dissipative snubber circuits, making it suitable for PEM electrolyzers operating over a wide output voltage range.

To enhance system integration, minimize energy conversion stages, and improve hydrogen production efficiency, multi-port DC–DC converters have emerged as a current research hotspot. Ref. [84] proposed a multi-port DC–DC converter topology (Figure 9e), in which Port 1 connects to renewable energy sources (RES), Port 2 interfaces with a fuel cell, and Port 3 connects to the electrolyzer. This configuration enables centralized control and integrated power conversion while optimizing the dynamic performance of hydrogen energy storage systems, thereby providing a feasible approach for the future development of DC–DC converters in water electrolysis applications.

### 3.2. AC-DC Converters

In renewable-energy-based hydrogen production systems with an AC bus structure, AC/DC converters supply power to the electrolyzer. These converters are classified into single-stage and two-stage types, depending on their structure.

**Figure 10:**
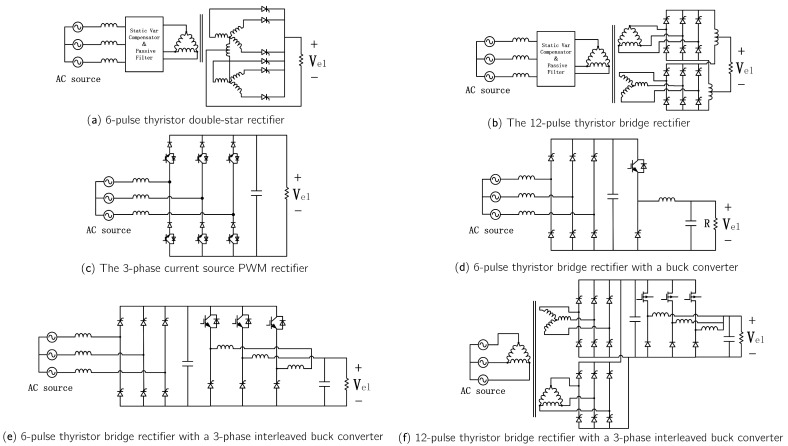
AC-DC converters for electrolyzer applications.

Early high-power industrial applications employed thyristor-based AC/DC converters, valued for their simplicity and low cost. For example, 6-pulse thyristor double-star rectifiers (Figure 10a) and 12-pulse thyristor bridge rectifiers (Figure 10b) have been successfully applied to power megawatt-scale alkaline electrolyzers [85]. Because of their technological maturity, thyristor topologies remain widely used in high-power industrial applications. However, they suffer from drawbacks, including high current ripple and poor power quality, particularly reactive power dependence on the trigger angle.

The advent of fully controlled semiconductor devices, such as Insulated Gate Bipolar Transistors (IGBTs) and Metal-Oxide-Semiconductor Field-Effect Transistors (MOSFETs), has enabled broader use of PWM converters in high-power applications. Current-source PWM rectifiers (Figure 10c) regulate a wide range of DC voltages through direct current control and fast dynamic response, making them suitable for low-voltage electrolyzer applications. In addition, current-source rectifiers exhibit high power factor, low input current distortion, and strong dynamic performance [86].

Alternatively, a wide bucking capability can be achieved by combining an AC/DC converter with a DC/DC converter. Muyeen et al. [87] employed a 6-pulse diode AC/DC rectifier combined with a Buck circuit (Figure 10d) for wind-energy-based hydrogen production. In this setup, multiple low-power electrolyzers are connected in parallel, each paired with a Buck circuit to mitigate wind power fluctuations by adjusting the number of active electrolyzers. This configuration offers fewer switching devices and lower cost; however, its output current ripple is constrained by inductance and switching frequency. To reduce output ripple, a three-phase interleaved Buck converter (Figure 10e) can be used instead. This topology offers fault-tolerant operation, high reliability, and reduced inductor size and current ripple.

For high-current applications, a 12-pulse thyristor bridge rectifier (Figure 10f) can replace the 6-pulse rectifier. This topology offers significant advantages in harmonic mitigation and current ripple reduction [88], yielding lower current THD and higher power factor. As a result, electrolyzer energy consumption decreases, though at the expense of increased cost and system size.

Single-stage AC/DC converters are typically employed in high-power industrial hydrogen production, such as for alkaline electrolyzers. In contrast, two-stage AC/DC converters are better suited for renewable-energy-based hydrogen production systems, owing to their wide output voltage regulation range and higher power factor.

### 3.3. Comparison and Analysis

High-performance electrolyzers require power supplies characterized by high power density, low current ripple, high efficiency, and strong operational reliability. Given the wide variety of converter topologies currently under investigation, establishing a unified performance evaluation standard remains a significant challenge. Table 4 summarizes and compares the efficiency, rated power, component count, application scale, and respective advantages and disadvantages of the aforementioned converter topologies. The reported efficiencies are extracted from experimental or simulation data presented in the cited literature. As the converter ratings and test conditions differ among studies, only representative operating points (rated or near-rated loads) are considered for efficiency comparison. Detailed test ranges are provided in the corresponding references.

Among non-isolated DC–DC converters, the classic Buck topology is simple and inexpensive but limited by high current ripple and low step-down capability, suiting only low-power PEM electrolyzers. Quadratic and double-quadratic Buck converters improve voltage reduction and dynamic response but at higher cost and complexity. Interleaved and three-level Buck topologies further reduce ripple and device stress, offering higher efficiency for high-current or high-voltage applications.

Isolated converters are essential for high-power and safety-critical systems. The half-bridge type provides moderate performance for medium-power alkaline electrolyzers, while the phase-shifted full-bridge achieves high efficiency via zero-voltage switching (ZVS). The push–pull converter is low-cost but less efficient, whereas resonant LLC and multi-port converters offer superior efficiency and integration flexibility, albeit with greater complexity and expense.

In AC–DC conversion, six-pulse thyristor rectifiers remain common in industrial alkaline electrolyzers for their simplicity, though they suffer from high harmonics and low power factor. Twelve-pulse versions mitigate harmonics, and PWM rectifiers improve power factor and dynamic control for smaller systems. Two-stage AC/DC converters further enhance power quality and voltage regulation, and three-phase interleaved designs provide improved redundancy and lower current ripple.

## 4. Control Strategy for Hydrogen Production Through Water Electrolysis

Implementing an effective control strategy for hydrogen production via water electrolysis is of critical importance. Such strategies not only enhance hydrogen pro-duction and efficiency but also ensure safe and stable system operation. Precise control of electrolysis voltage and current optimizes energy efficiency and extends equipment lifetime. Moreover, given the intermittency of renewable energy supply, the control system must adapt to continuous input fluctuations to maintain optimal operating conditions. Overall, efficient control strategies improve both the economic viability of hydrogen production and the environmental sustainability of the process [89]. Table 5 compares representative control strategies used in renewable energy driven electrolytic cell systems.

Han et al. [90] examined the use of a fuzzy PI (Proportional–Integral) control strategy in photovoltaic hydrogen production systems. In these systems, effective control is critical for enhancing energy conversion efficiency and stability. By integrating fuzzy logic with PI control, the strategy ensures stable performance under system complexity and uncertainty. Experimental results confirm that fuzzy PI-controlled photovoltaic hydrogen systems achieve stable operation across diverse conditions with high energy conversion efficiency.

Hossain et al. [91] introduced a novel dynamic control strategy that integrates Demand-Side Response (DSR) for the use of Proton Exchange Membrane Electrolyzers (PEMELs) in power system frequency regulation. By dynamically adjusting PEMEL power consumption in response to frequency fluctuations, the strategy maintains a re-al-time balance between generation and load. This approach enhances the system’s ability to regulate frequency deviations and improves the robustness and reliability of power grids with high shares of renewable energy.

Yodwong et al. [92] developed a new control algorithm for the Three-level Inter-leaved Buck Converter (TLIBC) to enhance efficiency in powering Proton Exchange Membrane Electrolyzers under renewable energy conditions. Built on an improved nonlinear sliding-mode control, the algorithm accommodates wide input voltage variations, balances input capacitors, reduces output ripple, and enhances fault tolerance. Experimental results show that it significantly outperforms conventional PI-based control under variable renewable inputs.

Xiong et al. [93] introduced a Multimode Self-Optimization Electrolysis Converting (MMSOEC) strategy to enhance the efficiency of alkaline water electrolyzers (AWEs). By combining direct current with pulsed power, the method improved efficiency by 15.4% under low-load conditions compared with conventional pure DC operation. This work addresses a major bottleneck in AWE-based hydrogen production, providing a practical and effective solution to boost efficiency and adaptability to renewable energy.

Guo et al. [94] presented a fault-tolerant operation method to improve the efficiency and stability of multiphase stacked interleaved Buck converters for green hydrogen production. The method eliminates output current ripple and maintains fault-tolerant operation during failures, thereby ensuring continuous and stable performance. It offers a valuable theoretical and practical foundation for the further development of green energy conversion technologies.

## 5. Optimal Combination Framework

**Figure 11:**
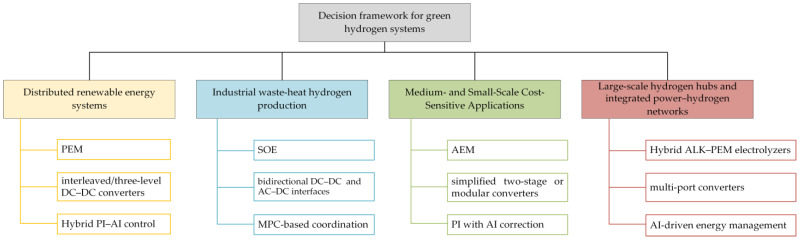
Decision Framework for Green Hydrogen Systems.

In recent years, extensive studies have focused separately on electrolysis technologies, power electronic topologies, and control strategies. However, single-dimension analyses often fail to address the systemic challenges encountered in complex application scenarios. This review argues that future optimization should not be confined to performance enhancement in isolated subsystems, but rather approached from a holistic “electrolyzer–power topology–control strategy” perspective, thereby forming tailored combination frameworks for specific application scenarios. Figure 11 shows the Decision Framework for Green Hydrogen Systems.

- Distributed renewable energy systems: The main challenge in this scenario is managing the fluctuating output of renewable sources such as solar and wind. Proton exchange membrane electrolyzers (PEMELs), with rapid start-up, wide power range, and strong transient response, are best suited for such conditions. Interleaved or three-level non-isolated DC–DC converters are preferred to suppress output current ripple, protect components, and maintain high efficiency with redundancy. At the control level, a hybrid PI–AI strategy is adopted: the inner PI loop ensures fast tracking, while the AI layer performs short-term prediction of renewable inputs to limit load ramp rates. This coordination enhances energy utilization and mitigates long-term degradation.
- Industrial waste-heat hydrogen production: This scenario utilizes stable, high-grade industrial waste heat. The solid oxide electrolyzer cell (SOEC), capable of directly using thermal energy, achieves much higher overall efficiency than low-temperature electrolysis. Paired bidirectional DC–DC and AC–DC interfaces provide flexible operation for both grid absorption and power feedback. Model predictive control (MPC) is employed to handle multi-variable thermal constraints, maintaining stable stack temperature and preventing material degradation, thereby ensuring long-term efficiency and reliability.
- Medium- and Small-Scale Cost-Sensitive Applications: For community energy storage or remote power supply, capital cost is the dominant factor. The anion exchange membrane electrolyzer (AEMEL) combines the low-cost advantage of alkaline systems with the compactness of PEM technology, offering the lowest cost per kilowatt. The power system typically uses a simplified two-stage topology—a diode rectifier followed by a non-isolated Buck converter—trading minor performance loss for reduced complexity and cost. A PI controller with lightweight AI-based adaptive tuning maintains high efficiency across wide load ranges while minimizing computational demand.
- Large-scale hydrogen hubs and integrated power–hydrogen networks: At the gigawatt scale, system design must balance cost, reliability, and grid interaction. A hybrid ALK–PEM configuration is typically employed: alkaline electrolyzers provide stable baseload operation, while PEM units handle peak regulation and frequency response. A multi-port DC–DC converter integrates PV, wind, and grid sources for flexible power distribution. A multi-layer control hierarchy is adopted, where an AI-driven energy management system (EMS) performs rolling optimization based on market and weather data to minimize hydrogen cost, while robust or sliding-mode control ensures fast and stable PEM operation under disturbances.

In summary, the optimal framework for future research lies not in pursuing isolated excellence in electrolysis, topology, or control, but in establishing customized combinations tailored to specific scenarios. This systematic perspective provides a structured roadmap for research efforts and offers more practical technical guidance for industrial deployment.

## 6. Application Prospect of Green Hydrogen Technology

**Figure 12:**
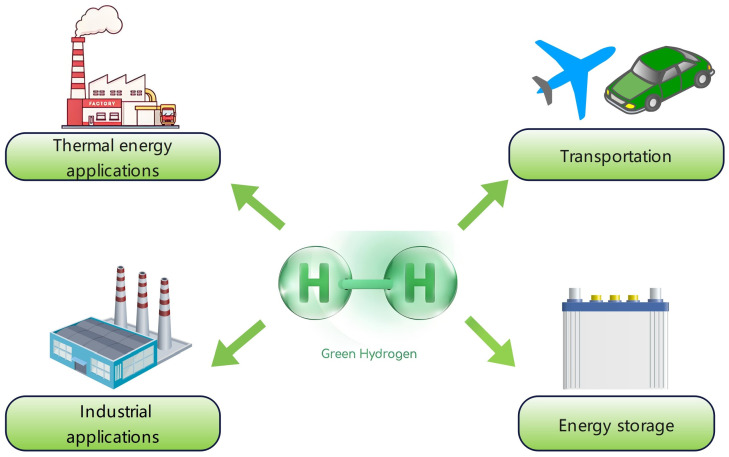
Prospects of green hydrogen energy applications.

As a clean and sustainable energy source, green hydrogen holds broad application prospects, as illustrated in Figure 12, spanning energy storage, transportation, industrial production, and thermal energy utilization. It is expected to play a pivotal role in the energy transition and in mitigating climate change. However, realizing these potentials requires continuous technological innovation, cost reduction, supportive policies, and an improved industrial chain to facilitate its large-scale adoption across diverse sectors [95].

### 6.1. Thermal Energy Applications

In green hydrogen production, the adoption of waste heat recovery technologies can significantly enhance energy efficiency, reduce production costs, and mitigate environmental impacts. Major applications include waste heat utilization in electrolysis-based hydrogen production, cogeneration systems, and thermal energy recycling systems [96]. In high-temperature industries such as steel, glass, cement, and ceramics, manufacturing processes often require heat exceeding 1000 °C. Green hydrogen can be directly used as a fuel in combustion heating systems to meet these demands, replacing natural gas or coal and substantially lowering the carbon footprint. Furthermore, in Combined Heat and Power (CHP) systems, green hydrogen enables the simultaneous generation of electricity and recycling of waste heat, thereby improving integrated energy efficiency. For low- and medium-temperature heat demands, it can be applied in district heating networks and hot water supply for buildings. When combined with fuel cells or thermal energy conversion devices, it reduces reliance on conventional combustion fuels, lowers carbon emissions, and provides a viable decarbonization pathway for urban energy systems. The comprehensive utilization of waste heat not only lowers production costs but also decreases carbon emissions, offering crucial support for the sustainable development of green hydrogen production [97].

### 6.2. Transportation

Green hydrogen shows considerable potential in the transportation sector, particularly in road transport and aerospace, which are major contributors to global greenhouse gas emissions. In road transportation, fuel cell vehicles generate electricity by combining hydrogen with oxygen, powering electric motors. Compared with traditional internal combustion engine vehicles, fuel cell vehicles offer the advantages of zero emissions and extended driving range, providing a cleaner and more sustainable solution for the transportation industry [98]. Similarly, in the aviation sector, hydrogen-powered aircraft present a promising decarbonization pathway, especially for long-haul flights that are heavily dependent on conventional jet fuel. The integration of hydrogen fuel cell technology into aircraft and spacecraft is expected to reduce emissions and enhance energy efficiency, steering the aerospace industry toward a cleaner and more sustainable future [99]. By replacing conventional fossil fuels with hydrogen, these industries can substantially reduce their carbon footprints, thereby contributing to the global transition toward cleaner energy systems. Hydrogen-powered aircraft and fuel cell vehicles thus represent transformative innovations that not only reduce air pollution but also advance long-term environmental and low-carbon transportation goals.

### 6.3. Industrial Applications

Green hydrogen is being increasingly adopted in industrial applications, holding significant potential for decarbonization in industrial production. For instance, in ammonia production—where hydrogen is a critical feedstock—the use of green hydrogen produced via electrolysis powered by renewable energy can make the process cleaner and more sustainable. In steel manufacturing, green hydrogen can replace coal-based reductants in the direct reduction of iron, substantially lowering CO_2_ emissions. Moreover, hydrogen can also be applied in processes such as glass production and food processing. Substituting green hydrogen for conventional fossil fuels reduces carbon emissions and environmental pollution in industrial processes, while also lowering production costs.

Promoting the use of green hydrogen in these applications can accelerate the transition of industrial production toward cleaner and more sustainable development. However, challenges such as technological maturity, cost competitiveness, and market adoption must be addressed to enable the widespread use of green hydrogen in industrial applications [100].

### 6.4. Energy Storage

The application of green hydrogen in energy storage is both promising and crucial, particularly for addressing the volatility and intermittency of renewable energy sources. As an efficient energy carrier, green hydrogen enables the storage of electrical energy through water electrolysis. When energy demand arises, the stored hydrogen reacts with oxygen to generate electricity, offering an effective solution for large-scale energy storage.

Diversified hydrogen storage is a critical enabler for large-scale green hydrogen deployment [101]. Hydrogen storage technologies encompass various physical and chemical methods tailored to different application needs and environmental conditions. Physical storage includes compressed, liquefied, and underground hydrogen storage, while chemical storage primarily involves metal hydrides and liquid ammonia. In parallel, developing high-value carbon materials such as graphite carbon derived from renewable lignocellulosic biomass (e.g., pine cones) for batteries and supercapacitors represents a complementary approach to enhance the sustainability of the overall energy storage ecosystem [102]. By integrating multiple hydrogen storage technologies, efficient storage and flexible utilization of green hydrogen can be realized across diverse application scenarios.

## 7. Conclusions

Despite the rapid progress of green hydrogen technologies, several fundamental scientific issues and technical bottlenecks remain to be addressed, constituting the major current research gaps. Alkaline electrolysis (ALK), though technologically mature, still struggles to adapt to the fluctuating nature of renewable energy; its dynamic response and electrolyte management strategies require further optimization. The core challenge for proton exchange membrane electrolysis (PEM) lies in the degradation and high cost of noble metal catalysts such as iridium. The development of highly active and stable low-iridium or iridium-free catalysts will be a key future direction. For anion exchange membrane electrolysis (AEM), the long-term chemical stability and mechanical durability of the membrane remain critical barriers to commercialization. Solid oxide electrolysis (SOEC), while offering high efficiency, is constrained by material degradation under high-temperature operation and high system complexity and cost, posing challenges for large-scale demonstration and industrial deployment.

Looking ahead, the sustainable development of green hydrogen technologies will depend on coordinated innovation across electrolyzer materials, power conversion, and system control. Efforts should focus on developing next-generation durable and cost-effective electrolyzer materials, optimizing the dynamic operation strategies of ALK systems, advancing modular and multi-port high-efficiency power conversion architectures, and creating adaptive intelligent control algorithms capable of smoothing renewable energy fluctuations. Moreover, exploration of synergistic operation mechanisms in integrated electro–hydrogen–thermal energy systems and the establishment of techno-economic evaluation criteria tailored to different application scenarios are of great importance. With continued technological breakthroughs, interdisciplinary collaboration, and strong policy support, green hydrogen is expected to progressively mature and ultimately provide essential support for building a clean, low-carbon, and sustainable global energy system.

## Figures and Tables

**Table 1:** Policies documents on hydrogen energy.

Countries	Release Date	Strategy/Planning
European Union	2020	A hydrogen strategy for a climate-neutral Europe [7]
The United States	2022	The U.S. National Clean Hydrogen Strategy and Roadmap [8]
Japan	2021	The 6th Strategic Energy Plan [9]
Australia	2024	National Hydrogen Strategy 2024 [10]
China	2021	14th Five-Year Plan for Renewable Energy Development [12]
China	2022	Medium and long-term plan for the development of hydrogen energy industry (2021–2035) [15]

**Table 2:** Nomenclature and Definitions of Key Terms, Abbreviations, and Metrics.

Category	Item	Full Name/Description
Abbreviations in the chapter of electrolysis technology	ALK (AE)	Alkaline Water Electrolysis
	PEM (PEME, PEMEL, PEMEC, PEMWE)	Proton Exchange Membrane Electrolysis (Cell)
	SOEC (SOE)	Solid Oxide Electrolysis Cell
	AEM (AEME, AEMWE)	Anion Exchange Membrane Electrolysis (Water Electrolysis)
	TRL	Technology Readiness Level
	HHV	Higher Heating Value
Chemical Elements and Compounds	NiFe LDH, CoFe LDH, NiCo_2_O_4_	Nickel-Iron Layered Double Hydroxide, Cobalt-Iron Layered Double Hydroxide, Nickel Cobaltite (Anode catalysts)
	PFSA	Perfluorosulfonic Acid
	YSZ	Yttria-Stabilized Zirconia
	ScSZ	Scandium-Stabilized Zirconia
	Ni-YSZ	Nickel—Yttria-Stabilized Zirconia (SOEC fuel electrode)
	LSM, LSCF	Lanthanum Strontium Manganite, Lanthanum Strontium Cobalt Ferrite (SOEC oxygen electrode)
	LST, LSCrM	Lanthanum Strontium Titanate, Lanthanum Strontium Chromium Manganite (Alternative fuel electrodes)
	QA	Quaternary Ammonium (AEM functional group)
	PAQ-x	Branched Poly(Arylene Quinuclidinium) membrane (Novel AEM)
	SEBS, PSU	Polystyrene-block-poly(ethylene-ran-butylene)-block-polystyrene, Polysulfone (AEM polymer backbones)
	HFM	Heteroatom-Free Microporous Framework (AEM material)
Other Key Terms	MEA	Membrane Electrode Assembly
	GDL	Gas Diffusion Layer
	PTL	Porous Transport Layer
	CL	Catalyst Layer
	THD	Total Harmonic Distortion
	TPB	Triple-Phase Boundary
	ALD	Atomic Layer Deposition
	PLD	Pulsed Laser Deposition

**Table 3:** Comparison of Different Electrolysis Technologies [16,17].

Type	ALK	PEM	SOE	AEM
Electrolyte	(20 × 10^40^ wt% KOH)	Polymer membrane (e.g., Nafion ^®^)	Yttria-stabilized Zirconia (YSZ)	Divinylbenzene (DVB) polymer carrier with 1 mol·L^−1^ KOH/NaOH
Cathode catalyst	Ni, Ni-Mo alloys	Pt, Pt-Pd	Ni/YSZ	Ni
Anode catalyst	Ni, Ni-Mo alloys	RuO_2_, IrO_2_	LSM/YSZ	Ni or Ni Fe Co alloy
Operating Temperature (°C)	65~100	20~80	500~1000	40~80
System efficiency (HHV; %) ^1^	68~77	62~77	89 (laboratory)	57~59
Technology Readiness Level (TRL)	Grade 9	Grade 9	Grade 7~8	Grade 6
Energy consumption (kWh/Nm^3^)	4.5~5.5	3.8~5.0	2.6~3.6	3.76~4.2
Lifetime stack (h)	20,000~90,000	50,000~100,000	20,000~40,000	>30,000
Degradation rates (μV h^−1^)	<3	<4	N/A	N/A
Current Density (A/cm^2^)	0.2~0.4	1.0~2.0	0.3~2.0	0.8~2.5
Voltage range(V)	1.4~3	1.4~3	1.0~1.5	1.4~2.0
H_2_ Purity	99.5–99.9998%	99.9–99.9999%	99.9%	99.9%
System response	Seconds	Milliseconds	Seconds	Seconds
Advantages	High stability High technology maturity Long operational lifetime	Higher electrolysis efficiency High current density Capable of high-pressure operation	High efficiency Low-cost catalyst	Low ohmic resistance Good gas separation No precious metal catalyst required
Disadvantages	Low current density Corrosive electrolyte Slow dynamic response	High membrane cost Limited durability	High operating temperature Poor durability High capital cost	Low maturity Poor long-term operational stability

^1^ All efficiencies are reported on an HHV basis. System efficiency of SOE includes system-level heat integration.

**Table 4:** Comparative analysis of different topologies.

Topology	Source	Efficiency ^1^	EL Type	Rated Power	Semiconductor Components	Scale ^2^	Characteristic
Figure 8a, [73]	DC	+	ALK	50 W	1Diode+1MOSFET	small	Few circuit devices and low cost; Simple control Limited step-down capability and large current ripple.
Figure 8b, [73]	DC	++	ALK	50 W	2 MOSFETs	Small	Few circuit devices and low cost; higher efficiency than classic buck Limited step-down capability and large current ripple
Figure 8c, [74]	DC	++	PEM	120W	3Diodes+1MOSFET	Small → Medium	Higher voltage step-down ratio than classic buck; Simple control High voltage stress on switching transistors
Figure 8d, [75]	DC	N/A	N/A	500W	6Diodes+2MOSFETs	Medium	Low voltage stress on switches; High step-down ratio Higher component count; Higher cost
Figure 8e, [76]	DC	+	PEM	400W	4 MOSFETs	Medium	Small current ripple; Simple control Limited step-down capability; low efficiency
Figure 8f, [77]	DC	N/A	N/A	1.5MW	4 MOSFETs	High	Reduced switch voltage stress; Suitable for high-voltage operation Complex topology; Higher design cost
Figure 9a, [80]	DC	N/A	N/A	1 kW	6 IGBTs	Medium	High step-down ratio, Smaller current ripple; Simple control Lower reliability
Figure 9b, [81]	DC	N/A	ALK	1 kW	2Diodes+4MOSFETs	Medium	High reliability; Good efficiency More complex control; Higher cost
Figure 9c, [82]	DC	++	ALK	5KW	2Diodes+2MOSFETs	Medium	Few switching devices and low cost; Simple control High switch voltage stress; Not suitable for high-power application
Figure 9d, [83]	DC	+++	N/A	7.2 kW	4Diodes+4MOSFETs	Medium	High efficiency; Zero-voltage switching across load range Complex resonant design
Figure 9e, [84]	DC	+++	PEM	1kw	4Diodes+8MOSFETs	Medium	High efficiency; Enables power integration and centralized control Complex topology; Higher cost
Figure 10a, [85]	AC	++	ALK	3 MW	6 Thyristors	High	High current output capacity; Suitable for low-voltage, high-current applications High harmonic distortion; Low power factor
Figure 10b, [85]	AC	++	ALK	3 MW	12 Thyristors	High	Lower harmonic distortion; Lower current ripple; Better power factor than 6-pulse More complex topology; Higher cost
Figure 10c, [86]	AC	+++	ALK	5 MW	6Diodes+6IGBTs	High	Low input current distortion; Wide DC voltage regulation range Lower efficiency under light load; More complex control
Figure 10d, [87]	AC	++	ALK	1.8 MW	7 Thyristors+1IGBT	High	Low cost; Controllable current output Current ripple limited by inductance and switching frequency
Figure 10e, [87]	AC	N/A	N/A	N/A	9 Thyristors+3 IGBTs	Medium →High	High reliability; Reduced ripple More complex design; Higher cost
Figure 10f, [88]	AC	+++	PEM	20MW	15Thyristors+3IGBTs	High	Suitable for large current applications; Low THD and high-power factor Complex topology; Higher cost

^1^ “+” → η < 90%; “++” → 90% ≤ η < 94%; “+++” → η ≥ 94%; ^2^ Small: Suitable for low-power electrolytic cells or experimental platforms; Medium: Suitable for kilowatt level PEM electrolyzer systems; High: Suitable for megawatt level industrial hydrogen production facilities (such as large alkaline or PEM electrolysis plants).

**Table 5:** Comparative analysis of different control strategies.

Control Strategies	Robustness	Computational Complexity	Dynamic Response
PI control	Low	Low	Moderate
Fuzzy PI control	Medium–High	Medium–High	High
MPC	High	High	Excellent
Sliding mode control	High	Medium	Excellent
Fault-Tolerant control	High	Medium	Good–Very Good
Multimode Self-Optimization	Medium	Low-Medium	Excellent at low loads

## Data Availability

No new data were created or analyzed in this study. Data sharing is not applicable to this article.
